# Gustatory Responsiveness of Honey Bees Colonized with a Defined or Conventional Gut Microbiota

**DOI:** 10.1264/jsme2.ME23081

**Published:** 2024-03-07

**Authors:** Shota Suenami, Masato Sato, Ryo Miyazaki

**Affiliations:** 1 Bioproduction Research Institute, National Institute of Advanced Industrial Science and Technology (AIST), Tsukuba 305–8566, Japan; 2 Computational Bio Big Data Open Innovation Laboratory (CBBD-OIL), AIST, Tokyo 169–8555, Japan; 3 Faculty of Life and Environmental Sciences, University of Tsukuba, Tsukuba 305–8572, Japan

**Keywords:** honey bee, gustatory responsiveness, behavior, gut microbiota, gut-brain axis

## Abstract

Gut microbes have many beneficial functions for host animals, such as food digestion and development of the immune system. An increasing number of studies report that gut bacteria also affect host neural function and behavior. The sucrose responsiveness of the western honey bee *Apis mellifera*, which harbors a characteristic gut microbiota, was recently reported to be increased by the presence of gut microbes. However, this responsiveness may vary depending on the experimental design, as animal behavior may be modulated by physiological states and environmental conditions. To evaluate the robustness of the effects of the gut microbiota on host gustatory responsiveness, we herein examined the sucrose responsiveness of honey bees colonized with a defined bacterial community or a conventional gut microbiota extracted from a field-collected bee. Although colonization was experimentally verified, sucrose responsiveness did not significantly differ among treatments after the 2- or 5-h starvation period. We concluded that the sucrose responsiveness of *A. mellifera* is not always affected by its gut microbiota. Therefore, host physiological conditions and environmental factors need to be considered when evaluating the impact of the gut microbiota on host neural function and behavior.

Many animals host a microbiome, and microbes are abundant in the digestive tract because it provides a stable and nutrient-rich environment. Host animals also gain benefits from their association with gut microbes. For example, gut microbes process food materials that are indigestible for a host animal, provide nutrient supplements that host animals cannot synthesize, and promote the development of the immune system (Sommer and Bäckhed, 2013). An increasing number of studies recently reported that various neural functions and behaviors of hosts are affected by gut microbes. In mouse studies, the sizes of some brain parts, such as the hippocampus, neuronal morphology in terms of dendritic length and spine density, and gene expression profiles in some brain parts were affected by the gut microbiota (Diaz Heijtz *et al.*, 2011; Luczynski *et al.*, 2016). The fecal transplantation of the gut microbiota from patients with autism spectrum disorder (ASD) into germ-free mice induced ASD-like behavioral phenotypes (Sharon *et al.*, 2019). Furthermore, the gut microbiota was shown to play a role in diet selection by mice and affected the plasma concentration of tryptophan, a precursor of neurotransmitter serotonin that is involved in foraging behavior (Trevelline and Kohl, 2022). These effects of the gut microbiota on host behaviors have also been observed in invertebrates. The walking speed of germ-free fruit flies was faster than that of conventional (CV) flies, which were inoculated with the gut microbiota of normally reared flies (Schretter *et al.*, 2018). Some commensal bacteria have been shown to reduce the appetite of flies fed amino acid-rich food, even though they were reared under amino acid-deprived conditions (Leitao-Goncalves *et al.*, 2017).

Despite these phenomena, host-microbe interactions have not yet been elucidated in detail due to a number of biological reasons. In mammals, gut microbial communities consist of hundreds of bacterial species (Lagkouvardos *et al.*, 2016), most of which are difficult to culture under laboratory conditions. Moreover, invertebrates often harbor simpler gut bacterial communities than mammals. In addition, the fruit fly *Drosophila melanogaster*, a model organism used in mole­cular biology, has a gut microbiota that easily changes depending on rearing conditions (Broderick *et al.*, 2014). Therefore, difficulties are associated with experimental manipulations of the gut microbial community.

The western honey bee *Apis mellifera* harbors a simple, but highly consistent gut microbiota. It consists of seven species (at 97% sequence identity of 16S rRNA) occupying more than 90% of the entire gut community (Kwong *et al.*, 2017; Suenami *et al.*, 2023). Five of the seven species, *Snodgrassella alvi* (*Betaproteobacteria*), *Gilliamella apicola* (*Gammaproteobacteria*), *Bifidobacterium asteroides* (*Actinomycetes*), *Bombilactobacillus* Firm-4, and *Lactobacillus* Firm-5 (*Bacilli*), have been recognized as core phylotypes that are prevalent in *A. mellifera* sampled across continents irrespective of collection sites (Moran *et al.*, 2012; Ellegaard *et al.*, 2020). Two other species, *Frischella perrara* (*Gammaproteobacteria*) and *Bartonella apis* (*Alphaproteobacteria*), are also *Apis*-specific bacteria, but are less prevalent than the core phylotypes. A technical advantage is that all these bacteria are culturable and may be inoculated into microbiota-depleted (MD) honey bees to reconstruct the gut bacterial community (Kešnerová *et al.*, 2017), which makes honey bees a versatile model for investigations on the effects of the gut microbiota on host behavior. Recent studies reported that some core bacteria increased sucrose responsiveness or improved cognitive function in honey bees (Zheng *et al.*, 2017; Zhang *et al.*, 2022a, 2022b). While these are striking findings, animal behavior and brain functions largely depend on host physiological states. These studies induced a starvation state before the sucrose responsiveness test by removing food from rearing conditions, suggesting that the hunger levels of the tested individuals were not the same, which may have resulted in variations.

In the present study, we investigated the robustness of the effects of the gut microbiota on the gustatory responsiveness of honey bees. We examined the sucrose responsiveness of honey bees colonized with a defined bacterial community or a gut microbiota extracted from a field-collected bee under a unified starvation period. The community structures of the experimentally manipulated gut microbiota were then assessed to verify our treatments.

## Materials and Methods

### Preparation of microbiota-depleted (MD) bees

MD bees were obtained from 3-4 different colonies of *A. mellifera* located at the National Institute of Advanced Industrial Science and Technology (Tsukuba, Japan) during the summer and autumn seasons of 2022. They were generated as described in previous studies (Kwong *et al.*, 2014; Kešnerová *et al.*, 2017). In brief, pupae at the P7–P8 stages (Ganeshina *et al.*, 2000) were removed from capped brood cells with tweezers and placed in a sterilized plastic dish with 1‍ ‍mL of sterilized 30% sucrose water. They were kept at 35°C with 75% relative humidity (RH). After 2 days, newly emerged MD bees were used in further colonization experiments.

### Preparation of gnotobiotic bees

To prepare gnotobiotic honey bees, which were colonized with a defined bacterial community, bacterial strains were selected from five core phylotypes of the honey bee gut microbiota: *S. alvi* wKB2 (Kwong and Moran, 2013), *G. apicola* ESL0297 (Quinn *et al.*, 2024), *B.* Firm-4 Hon2N (Olofsson *et al.*, 2014), *L.* Firm-5 ESL0186 (Kešnerová *et al.*, 2017), and *B. asteroides* ESL0170 (Kešnerová *et al.*, 2017). These strains were cultured under the following conditions: *S. alvi*, brain heart infusion-agar plate at 35°C under a 5% CO_2_ atmosphere; *G. apicola*, Columbia blood-agar with 5% sheep blood at 35°C under a 5% CO_2_ atmosphere; *B.* Firm-4, *L.* Firm-5, and *B. asteroides*, MRS-agar with 2% fructose and 0.144% L-cysteine hydrochloride monohydrate at 35°C under anaerobic conditions (Engel *et al.*, 2013). After 2 or 3 days of cultivation, each strain was collected with 2‍ ‍mL of sterilized 15% glycerol/PBS, and 20-μL aliquots of the suspension were stored at –80°C. The bacterial amount of each strain was estimated by the average of colony-forming units (CFU) in two aliquots.

Aliquot stocks of the five strains were mixed to 2.5×10^5^ CFU for each strain in 25% sucrose/PBS, resulting in approximately 5‍ ‍μL (4.42–4.89‍ ‍μL) of the mixture. Newly emerged MD bees were fed the mixture and reared in a plastic cage with sterilized 30% sucrose water, a pollen substitute, and synthetic queen pheromone to mimic in-hive conditions. Approximately 20 gnotobiotic bees were maintained in the cage for 8 days at 30°C with 60% RH and then used in further experiments. As a negative control, MD bees were fed 5‍ ‍μL of 25% sucrose/PBS (hereafter, PBS-treated bees) and reared under the same conditions.

### Preparation of conventional (CV) bees

A nurse bee was collected from a hive in 2022 and anesthetized on ice for 15‍ ‍min. The gut was dissected with tweezers under a Leica M125C binocular microscope (Leica). Malpighian tubules, the trachea, and midgut were removed, and the hindgut was placed into a 2-mL screw-capped tube with Ø1.0-mm glass beads and 1‍ ‍mL of sterilized 15% glycerol/PBS. The hindgut was then homogenized by bead beating (MicroSmash MS-100, TOMY) at 3,500‍ ‍rpm for 30 s. The homogenate was centrifuged at 600×*g* for 5‍ ‍min to remove debris. Twelve-microliter aliquots of the supernatant were stored at –80°C for colonization experiments, while 500‍ ‍μL of the supernatant was used for further DNA extraction.

An aliquot stock of the gut homogenate was diluted to become equivalent to 0.5×10^5^ copies of 16S rRNA gene μL^–1^ in 25% sucrose/PBS. Newly emerged MD bees were fed 5‍ ‍μL of the homogenate and reared under the same conditions as gnotobiotic bees.

### Sucrose responsiveness assay

The assay was conducted as previously reported (Scheiner *et al.*, 2013; Değirmenci *et al.*, 2018). Gnotobiotic bees, CV bees, and PBS-treated bees, prepared from the same hives for each, were anesthetized on ice for 15‍ ‍min and harnessed into 1.5-mL tubes using paraffin films. Bees were kept in an incubator at 30°C with 60% RH for 30‍ ‍min to wake them up. To control the hunger of bees, they were fed sterilized 30% sucrose water until they stopped showing proboscis extension. Bees were then returned to the incubator and kept for 2 or 5 h. In the sucrose responsiveness assay (Fig. S1), we randomly selected 5 gnotobiotic bees and 5 PBS-treated bees as a control, and tested their response to sucrose water at different concentrations of 0, 0.1, 0.3, 1, 3, 10, and 30%. Assay sessions were performed in ascending order from low to high sucrose concentrations. In each session, we tested a single concentration of sucrose. A PBS-treated bee was placed in the assay arena, familiarized for 10‍ ‍s, presented with 2‍ ‍μL of sucrose water for 5‍ ‍s, rested for 10‍ ‍s, and then released from the arena. After 5‍ ‍s, a gnotobiotic bee was then tested in the same manner. When all 5 gnotobiotic bees and 5 PBS-treated bees were tested alternately, the next session with another concentration of sucrose was started with an interval of 1‍ ‍min. Therefore, each bee was tested every 6‍ ‍min from low to high concentrations of sucrose. This was longer than the time (*i.e.*, 2‍ ‍min) during which a honey bee habituates or sensitizes to a stimulation (Scheiner *et al.*, 2013; Değirmenci *et al.*, 2018). We performed another round of the assay with another 5 gnotobiotic bees and 5 PBS-treated bees. The sucrose responsiveness of CV bees was tested with PBS-treated bees in the same manner. The responses of bees were recorded with a video camera.

If proboscis extension was observed when a given concentration of sucrose was presented, a gustatory responsive score (GRS) of one was given. Otherwise, GRS was zero. The sum of GRS was calculated for each bee and compared across treatments. When bees showed proboscis extension before the presentation of sucrose water, they were considered to have learned the experimental set-up and were omitted from the ana­lysis.

After the sucrose responsiveness assay, bees were anesthetized on ice and their abdomens were separately placed into 1‍ ‍mL of absolute ethanol and stored at –80°C for DNA extraction.

### DNA extraction and quantification by quantitative PCR (qPCR)

The hindguts of the bees analyzed in the sucrose responsiveness assay were dissected in the same manner as that described above. The hindgut was placed into Lysing Matrix E (MP-Biomedicals), and DNA extraction was performed using the FastDNA Spin Kit for Soil (MP-Biomedicals) according to the manufacturer’s instructions. Homogenization was conducted using MicroSmash MS-100 at 3,500‍ ‍rpm for 90‍ ‍s twice with an interval of 1‍ ‍min on ice. Extracted DNA was dissolved in 50‍ ‍μL of elution solution. We mixed 500‍ ‍μL of the hindgut homogenate of the nurse bee (see above) with 478‍ ‍μL of sodium phosphate buffer from the kit and extracted DNA, as described in the instructions. DNA concentrations were measured using the Qubit dsDNA HS Assay Kit (ThermoFisher Scientific).

Extracted DNA was diluted 100-fold with DNase/RNase-free water and used as the template for qPCR. The V3–V4 region of the bacterial 16S rRNA gene was amplified with the following universal primers: forward (5′-ACTCCTACGGGAGGCAGCAGT-3′) and reverse (5′-ATTACCGCGGCTGCTGGC-3′) (Ellegaard *et al.*, 2020). Each reaction was performed in triplicate in a total volume of 10‍ ‍μL, containing 5‍ ‍μL of 2×TB Green premix Ex Taq II (TaKaRa), 0.2‍ ‍μL of Rox reference dye II, 0.2‍ ‍μM of each primer, 3.4‍ ‍μL of distilled water, and 1‍ ‍μL of the DNA template. qPCR was conducted using QuantStudio3 (Applied Biosystems) with the following program: denaturation at 95°C for 30‍ ‍s and 40 amplification cycles at 95°C for 5‍ ‍s and 60°C for 30 s. Melting curves were generated after each run with the default setting. To quantify the absolute copy number of the target 16S rRNA gene sequence in samples, standard curves were generated by the serial dilution of the target sequence, which was obtained by PCR with pMD20 T-Vector (TaKaRa) containing the sequence as a template. Significant differences in the copy number obtained between different treatments and starvation periods were evaluated by the pairwise Wilcoxon rank-sum test with the Benjamini-Hochberg multiple p-value adjustment method.

### 16S rRNA gene amplicon sequencing and community ana­lysis

The V4 region of the bacterial 16S rRNA gene was amplified using 0.5‍ ‍ng of the DNA template and the universal primers 515F (5′-GTGCCAGCMGCCGCGGTAA-3′) and 806R (5′-GGACTACHVGGGTWTCTAAT-3′). PCR reactions were performed in 10‍ ‍μL with KOD FX Neo DNA polymerase (TOYOBO) at an annealing temperature of 50°C for 30 cycles. PCR products were purified using VAHTS DNA Clean beads (Vazyme). Five nanograms of the first PCR product was used for subsequent short PCR with Illumina barcoded primers for 10 cycles. The second PCR products were purified with VAHTS DNA Clean beads, and their quality was checked with the QuantiFluor dsDNA System (Promega) and Fragment Analyzer (Agilent Technologies). Sequencing was performed by the Illumina MiSeq 2×300 bp pair-end platform. An average of 44,379 pairs of reads were obtained per sample.

### Analysis of the microbial community composition

We used QIIME2 2023.5 (Bolyen *et al.*, 2019) to process sequencing data and conduct a community ana­lysis. Raw sequencing reads were processed for an ana­lysis of the microbial composition by denoising primers and low-quality sequences with a read length <300 using DADA2 and removing chimera detected by VSEARCH. Taxonomic assignment was performed against the 16S rRNA sequence database in SILVA 138 SSU (Yilmaz *et al.*, 2014). Based on sequence similarities (99%), taxonomic assignment to the species level was performed for all 16S rRNA sequences. Sequences assigned as mitochondria or chloroplasts were filtered and excluded from subsequent ana­lyses. In cases where clusters were not assigned at the species level due to the lack of sequences or redundant sequences in the database, the taxon was assigned at the genus level. We calculated beta-diversity with Bray-Curtis, Jaccard, and weighted and unweighted UniFrac distances based on the OTU reads of taxa at the species level using the QIIME2 core metric pipeline. Each beta-diversity value was visualized by a principal coordinate ana­lysis (PCoA). The significance of differences between treatment groups (PBS-treated, gnotobiotic, or CV) and starvation periods (2 or 5‍ ‍h), and these interaction terms were tested by a multi-factor permutational ana­lysis of variance (PERMANOVA) using the Adonis plugin.

## Results

### Sucrose responsiveness of gnotobiotic and CV honey bees

To investigate whether the gut microbiota affected the sucrose responsiveness of honey bees, we prepared gnotobiotic honey bees with five bacterial strains: *S. alvi*, *G. apicola*, *B. mellis* (Firm-4), *L. kullabergensis* (Firm-5), and *B. asteroides*, which are common and dominant phylotypes in the honey bee gut microbiota (Kwong and Moran, 2016; Kwong *et al.*, 2017). We also prepared CV honey bees inoculated with the gut microbiota of a field-collected honey bee, harboring more diverse bacterial species, including minor populations, than gnotobiotic bees. As a negative control, MD bees were treated with sterilized PBS/sucrose water. After 8 days of rearing, the survival rates of gnotobiotic and PBS-treated groups were both 98%, while that of the CV group was 94%.

**Fig. 1.**
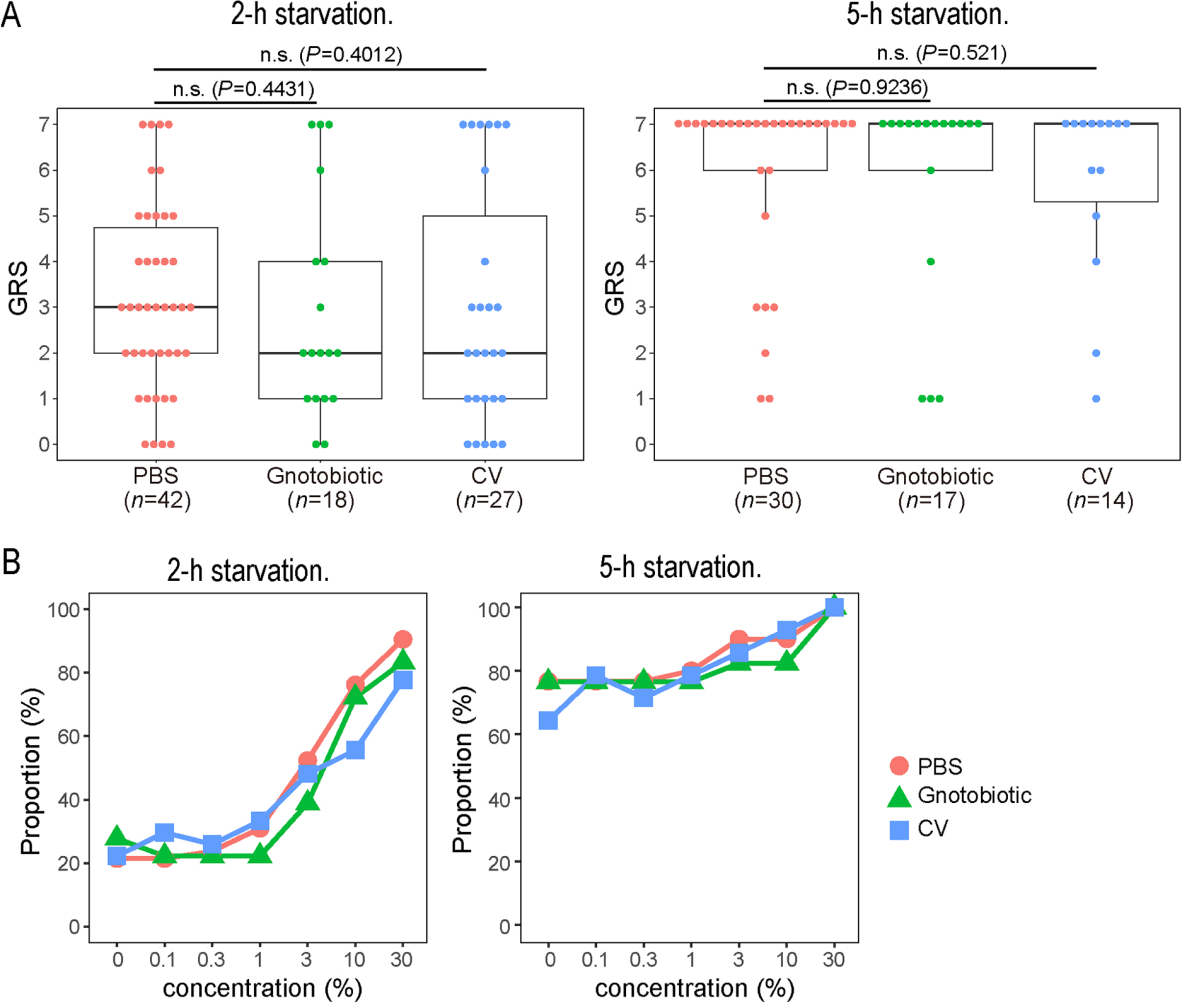
Sucrose responsiveness of honey bees. (A) Gustatory responsive scores (GRS) of PBS-treated bees, gnotobiotic bees, and CV bees after a 2-h (left panel) or 5-h (right panel) starvation period. Statistical tests were performed to compare differences between PBS-treated bees and gnotobiotic or CV bees. n.s., not significantly different (the Mann-Whitney U test, *P*>0.05). (B) Percentage of bees responding to sucrose water at different concentrations after a 2-h (left panel) or 5-h (right panel) starvation period. The same dataset as in A was used.

Since sucrose responsiveness is generally affected by the appetite of bees (Mayack *et al.*, 2019), we tested their responsiveness 2 or 5‍ ‍h after feeding 30% sucrose water (*i.e.*, 2-h or 5-h starvation period) to reduce variability in hunger among individual bees. As expected, GRS was higher in 5-h starved bees than in 2-h starved bees (Fig. 1A). This result indicated that the starvation period was sufficient to make bees hungry. However, no significant differences were observed between PBS-treated bees and gnotobiotic or CV bees irrespective of the starvation periods (the Mann-Whitney U test, *P*>0.05). Using the same dataset, we also calculated the percentage of bees responding to water with different concentrations of sucrose. In both starvation periods, the higher the concentration of sucrose, the more bees responded (Fig. 1B). The baseline of the ratio was higher in 5-h starved bees than in 2-h starved bees, which was consistent with previous findings (Mayack *et al.*, 2019). However, no significant differences were noted between PBS-treated control and gnotobiotic or CV bees under both starvation conditions. These results suggest that the gut bacteria did not affect the sucrose responsiveness of honey bees, at least under these experimental settings.

### Gut bacterial communities of gnotobiotic and CV honey bees

**Fig. 2.**
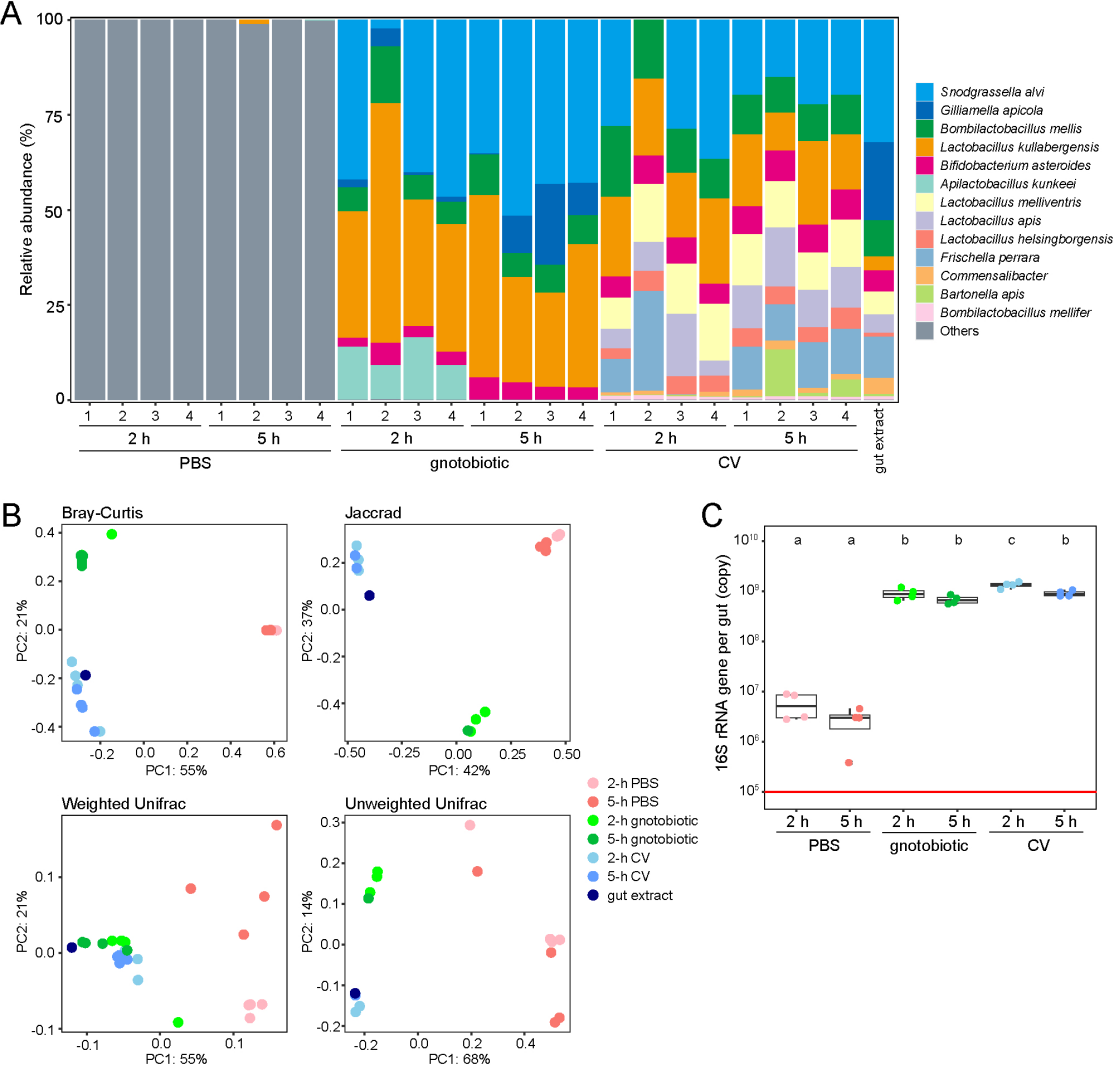
Gut bacterial community structures of honey bees. (A) Microbial compositions of individual honey bees. Relative abundance of microbial taxa in the hindgut of individual honey bees with different treatments (PBS, gnotobiotic, or CV) and starvation periods (2‍ ‍h or 5‍ ‍h) and that in the input gut extract are shown on stacked bar plots. Microbial taxa detected in PBS-treated bees only are categorized as “Others”. (B) PCoA plots representing the beta-diversity of different treatments and starvation periods. Each panel indicates a PCoA plot based on Bray-Curtis (left top), Jaccard (right top), weighted UniFrac (left bottom), and unweighted UniFrac (right bottom) distances. In PCoA plots, each circle represents the data of an individual sample (*n*=4 per group and *n*=1 for the input gut extract). In all indexes, differences in treatment had a significant effect on the microbial composition: Bray-Curtis, *R*^2^=0.858 and *P*<0.001; Jaccard, *R*^2^=0.917 and *P*<0.001; weighted UniFrac, *R*^2^=0.708 and *P*<0.001; unweighted UniFrac, *R*^2^=0.945 and *P*<0.001. Note that the starvation period and interaction term of the treatment and starvation period did not significantly affect the microbial composition. (C) Absolute copy numbers of the 16S rRNA gene in individual honey bee guts. The copy number was measured by qPCR using the same dataset as in A. The detection limit is indicated as a red line. Different letters indicate significant differences between different treatments and starvation periods (*P*<0.05, the pairwise Wilcoxon rank-sum test with the Benjamini-Hochberg multiple *P*-value adjustment method).

To confirm the colonization of gut microbes in gnotobiotic and CV bees tested in the sucrose responsiveness assay, we investigated the community structures of their gut microbiota by sequencing 16S rRNA genes. We randomly selected 4 bees in each treatment (*i.e.*, PBS-treated, gnotobiotic, and CV bees) and extracted DNA from their guts. All five core gut bacteria (*S. alvi*, *G. apicola*, *B.* Firm-4, *L.* Firm-5, and *B. asteroids*) that we inoculated into bees were detected in all gnotobiotic samples irrespective of the starvation periods (Fig. 2A). *Apilactobacillus kunkeei*, an opportunistic non-core species often found in honey bees and hives, was detected in 2-h starved gnotobiotic bees. The microbial compositions of CV bees were similar to that of the input gut homogenate irrespective of the starvation periods (Fig. 2A). This was further supported by the beta-diversity ana­lysis (Fig. 2B). Although the spontaneous infection of bacteria, which were unrelated to the honey bee gut microbiota, was detected in PBS-treated bees (Fig. 2A and S2, Table S1), their absolute abundance were significantly lower (less than 1%, *P*<0.05) than those of entire communities in gnotobiotic and CV bees (Fig. 2C), and were at a similar level (approximately 10^5^–10^6^ 16S rRNA gene copies per gut) to those of MD bees in previous studies (Liberti *et al.*, 2022) (Cabirol *et al.*, 2023 A defined community of core gut microbiota members promotes cognitive performance in honey bees. *bioRxiv* doi: https://doi.org/10.1101/2023.01.03.522593). These results were sufficient to confirm that gnotobiotic and CV bee samples were colonized almost uniformly with the expected bacterial communities.

## Discussion

The present results showed that sucrose responsiveness was indistinguishable between PBS-treated bees and gnotobiotic or CV bees after 2-h and 5-h starvation periods, indicating that the gut microbiota of honey bees did not have a significant impact on the host response and sensitivity to sucrose under these experimental settings. On the other hand, previous studies reported that the presence of the gut microbiota or a mono-association with *G. apicola*, *B.* Firm-4, or *L.* Firm-5 enhanced sucrose responsiveness (Zheng *et al.*, 2017; Zhang *et al.*, 2022a). Disparities between the present results and previous findings may be attributed to differences in rearing conditions. In previous studies, the starvation period was defined as the rearing time without food before the sucrose responsiveness assay. However, fasting states before starvation vary among bees, resulting in different states of samples at the time of the assay. We found that 5-h starved bees exhibited higher sucrose responsiveness than 2-h starved bees. Therefore, we manually satiated individual bees with sucrose and then subjected them to starvation. Another difference in experimental settings is that the honey bees used in previous studies were reared in the absence of the queen pheromone, to which honey bees are generally exposed in their nests. A previous study showed that the queen pheromone reduced the sucrose responsiveness of honey bee workers (Pankiw and Page, 2003). Although the presence of the queen pheromone in the present study may have masked the potential effects of gut bacteria, this condition was more reflective of the natural conditions of in-hive bees. We hypothesized that the gut microbiota affects the activities of foragers, which go outside and are less exposed to the queen pheromone. If this is the case, food consumption by in-hive bees may be suppressed by the queen pheromone, while food gathering by foragers may be enhanced by the gut microbiota.

Another plausible explanation for discrepancies between the present results and previous findings is differences in the profiles of the gut microbiota. We confirmed that the community structures and bacterial abundance of CV bees were consistent among samples in this study; however, they were not examined in previous studies (Zheng *et al.*, 2017; Zhang *et al.*, 2022a). In colonization experiments, the final community structures of the honey bee gut microbiota need to be inspected because they may vary among samples depending on rearing conditions, diet, and biological variations. The establishment of the gut microbiota requires a specific period of time (approximately 5–7 days), which may vary among individual bees (Kešnerová *et al.*, 2017). Even after a sufficient time for its establishment, some individual bees occasionally lack some gut microbes, such as *Gilliamella* in the present study (Fig. 2A) and a previous study (Liberti *et al.*, 2022), when a conventional gut microbiota is experimentally introduced. A recent study reported that some diets affected not only the diversity and absolute abundance of the gut microbiota in honey bees, but also the expression of the insulin receptor genes *InR1* and *InR2*, which are involved in sucrose responsiveness (Powell *et al.*, 2023).

MD bees that emerged in the lab sometimes contained spontaneous microbes. When pupae were removed from capped brood cells, some microbes may have been transferred from the hive environment through the surface of pupae. Some taxa (*e.g.*, *Lactobacillus*, *Streptococcus*, *Pediococcus*, *Wissella*, and *Bacillus*) detected in MD bees in the present study have been also found in bee hive environments, such as hive-stored beebread, pollen, and nectar (Anderson *et al.*, 2013, 2014; Kahraman-Ilikkan, 2023). There was also a small amount of other environmental bacteria in MD bees, which may have been due to contamination as a result of bee manipulations. In previous studies, MD bees generally harbored less than 10^5^–10^7^ copies of the 16S rRNA gene per gut (Kešnerová *et al.*, 2017; Liberti *et al.*, 2022) (Cabirol *et‍ ‍al.*, 2023 *bioRxiv* doi: https://doi.org/10.1101/2023.01.03.522593). MD bees in the present study may have had a slightly high number (*i.e.*, 5.7×10^6^ copies in 2-h starved bees and 2.7×10^6^ copies in 5-h starved bees), which was still within the range reported in previous studies.

Consistent with the present results, a recent study clearly demonstrated that gut microbiota compositions did not affect the sucrose responsiveness of *A. mellifera* (Cabirol *et al.*, 2023 *bioRxiv* doi: https://doi.org/10.1101/2023.01.03.522593). They found no significant difference in sucrose responsiveness after an 18-h starvation period between MD bees and mono-colonized bees with the five core microbes nor bees colonized with a defined community (similar to gnotobiotic bees in the present study). These findings are reliable in that the compositions and abundance of the gut microbiota in samples were experimentally examined. On the other hand, the methods for the sucrose responsiveness assay slightly differed among previous studies (Table S2). Not only the method and the duration of starvation of bees, but also the criteria for evaluating responsiveness vary: (i) selecting response thresholds (the lowest sucrose concentration to which bees show proboscis extension) (Zheng *et al.*, 2017; Zhang *et al.*, 2022a); (ii) measuring response numbers to all‍ ‍sucrose concentrations, except 0% solution (Cabirol *et‍ ‍al.*, 2023 *bioRxiv* doi: https://doi.org/10.1101/2023.01.03.522593); (iii) measuring response numbers to all sucrose concentrations, including 0% solution (this study). Since these methodologies were based on previous studies (Page *et al.*, 1998; Scheiner *et al.*, 2003, 2004, 2013; Mengoni Gonalons and Farina, 2015; Değirmenci *et al.*, 2018), they were considered to be fundamentally sound for assessments of sucrose responsiveness and, thus, other factors (*e.g.*, the hunger levels of individual bees and rearing conditions) exerted stronger effects on behavior.

In conclusion, the sucrose responsiveness of the honey bee *A. mellifera* is not always impacted by its gut microbiota. While the neural function and behavior of animals may be affected by gut microbes, animal behavior is generally sensitive to their physiological states and environmental conditions, leading to different consequences.

## Citation

Suenami, S., Sato, M., and Miyazaki, R. (2024) Gustatory Responsiveness of Honey Bees Colonized with a Defined or Conventional Gut Microbiota. *Microbes Environ ***39**: ME23081.

https://doi.org/10.1264/jsme2.ME23081

## Supplementary Material

Supplementary Material 1

Supplementary Material 2-1

Supplementary Material 2-2

Supplementary Material 3
