# Supplementary material for: Gustatory Responsiveness of Honey Bees Colonized with a Defined or Conventional Gut Microbiota
Source: Microbes Environ. 2024 Mar 7;39(1):ME23081. doi: 10.1264/jsme2.ME23081 (PMC10982108; doi:10.1264/jsme2.ME23081)
Supplement: Supplementary file 1 — Supplementary Material 1 [file 39_23081_s1.pdf]

Supplementary materials for:

Gustatory responsiveness of honey bees colonized with a defined or conventional gut microbiota

Shota Suenami, Masato Sato, Ryo Miyazaki*

* Correspondence: ryo.miyazaki@aist.go.jp

Contents:

Fig S1. Experimental procedure of sucrose responsiveness assay.

Fig S2. Detailed microbial compositions of individual honey bees.

Table S1. ASVs, number of reads, and estimated lineages for each sample.

Table S2. A summary of the behavioral test methodologies used in relevant studies.

Movie S1. Representative movie of honey bees responding or not responding to sucrose stimulation. (The movie is provided in the separate file)

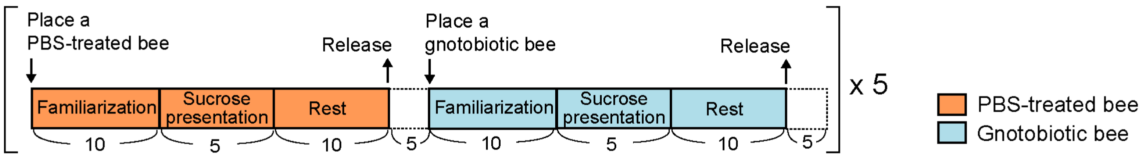

Fig S1. Experimental procedure of sucrose responsiveness assay. An assay session with PBS-treated and gnotobiotic bees is illustrated. Five bees from each are tested alternately with a given concentration of sucrose. After a 1-min interval, the next session with another concentration of sucrose is started. Assay sessions are carried out in ascending order from low to high sucrose concentrations. CV bees are tested with PBS-treated bees in the same way. Numbers below boxes indicate time (sec).

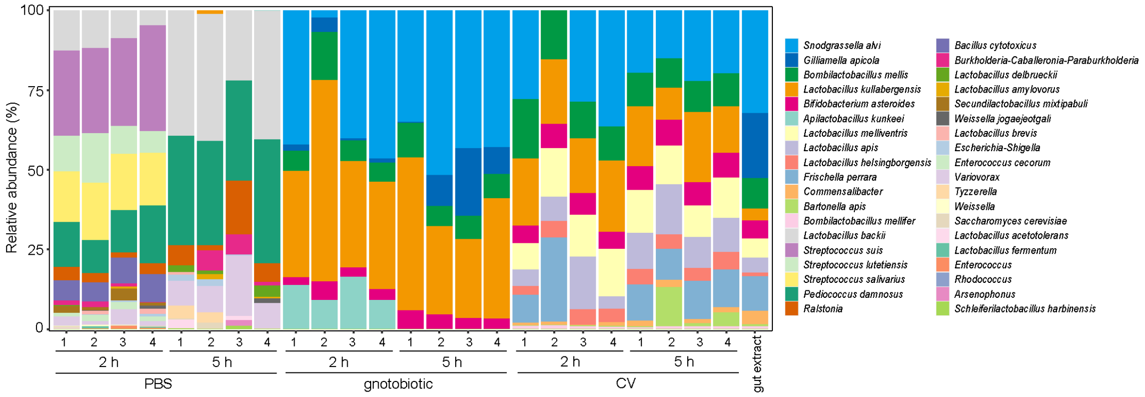

Fig S2. Detailed microbial compositions of individual honey bees. Same as Fig. 2A, although detailed taxa categorized as “Others” in Fig. 2A are shown.
